# Compounds of Marine Origin with Possible Applications as Healing Agents

**DOI:** 10.3390/md23010005

**Published:** 2024-12-26

**Authors:** Nektaria-Ioanna Karma, Fotini Mellou, Panagoula Pavlou, Angeliki Siamidi, Athanasia Varvaresou

**Affiliations:** 1 Division of Aesthetics and Cosmetic Science, Department of Biomedical Sciences, University of West Attica, 28 Agios Spyridonos Street, GR-12243 Egaleo, Greece; ak212203@uniwa.gr (N.-I.K.); ppavlou@uniwa.gr (P.P.); avarvares@uniwa.gr (A.V.); 2 Laboratory of Chemistry, Biochemistry and Cosmetic Science, Department of Biomedical Sciences, University of West Attica, 28 Agios Spyridonos Street, GR-12243 Egaleo, Greece; 3 Section of Pharmaceutical Technology, Department of Pharmacy, School of Health Sciences, National and Kapodistrian University of Athens, Panepistimioupoli-Zografou, GR-15784 Athens, Greece; asiamidi@pharm.uoa.gr

**Keywords:** innovative skin wound treatments, marine organisms, active marine ingredients, wound healing, wound dressing

## Abstract

It is well established that marine organisms consist of a great variety of active compounds that appear exclusively in the marine environment while having the ability to be vastly reproduced, irrespective of the existing conditions. As a result, marine organisms can be used in many scientific fields, including the ones of pharmaceutics, nutrition, and cosmetic science. As for the latter, marine ingredients have been successfully included in cosmetic formulations for many decades, providing numerous benefits for the skin. In the present review, the contribution of marine compounds in wound healing is thoroughly discussed, focusing on their role both as active ingredients in suitable formulations, designed to contribute to different stages of skin regeneration and restoration and also, indirectly, as a tool for facilitating wound closure as part of a wound dressing. Additionally, the advantages of these marine ingredients are presented, as well as ways of incorporating them effectively in formulations, so as to enhance their performance. Numerous studies have been referenced, showcasing their efficacy in wound healing. Finally, important data in regard to their stability, limitations, and challenges to their use, safety issues, and the existing legislative framework are extensively reviewed.

## 1. Introduction

Wounds are caused by physical injuries, resulting from the rupture of a living tissue’s genetic, anatomical, or functional continuity, either externally or internally. The appearance of a wound arises from a physical, chemical, thermal, microbial, or immune attack on the tissue. An open wound occurs when the skin is cracked, cut, or perforated, while a closed wound appears due to exercising force on the skin. Burns occur after the effects of fire, heat, chemicals, electricity, or solar radiation. The classification of wounds into acute and chronic is based on their extent and the healing period. Acute wounds heal quickly, with healing times ranging from days to weeks, but chronic wounds can take months to years to heal completely [1,2]. Wound healing is a complex physiologic process in which the body attempts to replace destroyed and damaged tissue with newly generated tissue and restore the skin’s barrier functions. Effective wound healing is one of the most desirable and impactful medical outcomes.

Natural compounds have been widely used for centuries to treat wounds. Currently, research focuses on the role of natural products in wound healing. Natural compounds constitute important active ingredients for wound healing, which can be found in both plants and animals [3]. A new direction for wound treatments is the use of natural compounds derived from marine resources. The marine environment offers multiple sources of biomaterials for wound healing and tissue regeneration. Marine biomaterials, although confirmed to be applicable, are still an underused resource [4].

**Figure 1:**
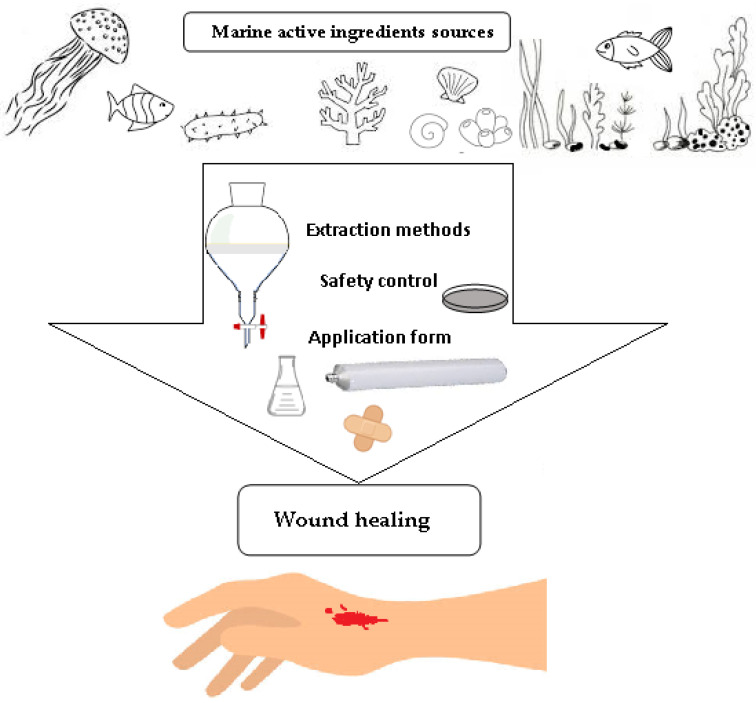
Marine-based bioactive compounds as potential healing agents.

In this review article, an overview of normal wound-healing phases (namely, hemostasis, inflammation, proliferation, and remodeling) is provided, and the factors that influence the healing mechanism are also highlighted. This study also consolidates recent findings proving the beneficial effectiveness of bioactive molecules derived from marine ecosystems in wound healing. Moreover, difficulties and problems regarding the stability of these substances are noted, while ways to improve them are proposed. In addition, issues and concerns related to the safety of use and the potential toxicity of chemical compounds from the marine environment are mentioned, as well as the ecological impact caused by the use of these ingredients. Figure 1 presents a graphical representation, allowing for visualization of the objective of this review article. Finally, emphasis is placed on the legislative framework governing these active substances to allow their incorporation into final products. Scientific databases such as PubMed, Science Direct, Scopus, and Web of Science were used for collecting scientific articles and chapters.

## 2. Skin Wound Healing (Mechanisms, Factors, and Treatment)

**Figure 2:**
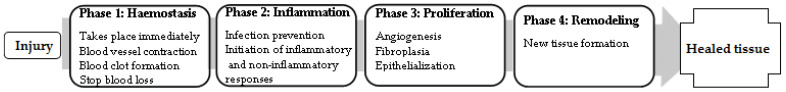
The four phases of wound healing.

Healing is defined as the natural mechanism of repair after injury. Normal wound healing is a highly sequential process of skin barrier function restoration and occurs in four distinct, overlapping, and sequential phases, including hemostasis, inflammation, proliferation, and tissue remodeling (Figure 2). Hemostasis begins immediately after injury and could last for several hours. As an immediate response to limit blood loss after injury, the blood vessels’ smooth muscle contracts via vasoconstrictors released by the damaged endothelial cells. This is followed by blood clot formation. Following injury, the inflammatory phase begins within minutes, peaks in two to three days, and can last one to two weeks, depending on the extent of the injury. The primary functions of the inflammatory phase during wound healing are to protect wounds against invading pathogens and to initiate the subsequent inflammatory and non-inflammatory responses needed for proper healing. The proliferative phase, also known as the new tissue regeneration phase, begins approximately three days after injury and lasts for weeks, laying a framework for repair. The main events during the proliferative phase are angiogenesis, fibroplasia, and epithelialization. The remodeling phase begins two to three weeks following injury and can last up to a year or even longer. Matrix maturation and tissue remodeling depend on the balance between the degradation of extracellular matrix components in granulation tissue and their replacement by connective tissue components, namely collagen I [2].

After causing damage, the restoration to the original state is never complete. In mammals, there is no possibility of organ regeneration. Injury to bones and liver are excluded. All human tissues, except teeth, can be set into a healing state, through a common procedure aimed at limiting damage and restoring the affected tissues structurally and functionally.

The healing process consists of a complex mechanism influenced by a variety of factors and compounds. Healing is classified into epidermal and in-depth healing. Healing of epidermal wounds is an important step in wound restoration, due to the necessary function of the epidermis as a physical, chemical, and bacterial barrier.

The factors that influence healing are local, such as temperature infection of the skin and oxygenation, but also systemic, such as age, diabetes, sexual hormones, genetic factors, and autoimmune diseases.

The majority of conventional skin wound treatments include the use of non-steroidal anti-inflammatory drugs (NSAIDs), immunomodulating agents, and topical corticosteroids, aiming at reducing inflammation. However, these medicines may have a negative effect on healing, as well as side effects such as atrophy, osteoporosis, obesity, and glaucoma [5,6].

## 3. Natural Products for Skin Wound Healing—Marine Ingredients

Products of natural origin have been used for their antioxidant, anti-inflammatory, antiviral, and regenerative properties in wound repair. Plant extracts, as well as substances of plant origin, contain ingredients that enhance re-epithelialization and collagen production, thus contributing to healing.

A recent trend in the field of cosmetics is the search for ingredients from the marine environment, due to their unique chemical and biological properties. Marine ingredients are not only used as viscosity regulators but also as active ingredients with a variety of actions, including hydrating, antimicrobial protection—cosmetic product preservation, Ultra Violet (UV) protection, coloring, bleaching, antioxidant, anti-aging, and anti-acne applications.

The “traditional” active substances of natural origin of dermocosmetics present a plethora of beneficial properties for the health of the skin, hair, nails, and also at the cellular level. However, they show some limitations due to the time required for the cultivation of plants and the chemical composition of the soil, which varies by season and region since plants do not thrive year-round and everywhere. On the contrary, marine plant and animal organisms, as well as marine micro-organisms, experience rapid and rich growth with high economic efficiency while producing chemicals not found in terrestrial environments. Bioactive ingredients, suitable for use in cosmetic science, e.g., vitamins and minerals, as well as fluorothanins, polysaccharides, carotenoids, and other pigments, chito-oligosaccharide derivatives (COS), enzymes, and peptides, have been isolated from organisms such as macro- and micro-algae. However, there is concern regarding the increasing extraction of marine components and the use of unsustainable methods to isolate them, thereby further disrupting the sustainability of aquatic ecosystems [7,8].

## 4. Ingredients from Sea Organisms

Marine life is diverse, including sponges, corals, algae, and other forms of organisms that harbor numerous bioactive substances. These can be employed in various sectors, such as the pharmaceutical and cosmetic industries. Scientific research on various applications of active ingredients extracted from these organisms has become increasingly prevalent.

### 4.1. Sea Cucumbers

Sea cucumbers are marine invertebrates that belong to the homodegradation “Holothuroids” and can be found on the seabed all over the world. Sea cucumbers are rich in chemical compounds with biological action, such as saponins, chondroitin, sulfates, collagen, vitamins, amino acids, phenols, triterpenic glycosides, carotenoids, bioactive peptides, trace elements, fatty acids, and gelatin. They exhibit anti-cancer, antioxidant, antimicrobial, anticoagulant, and neuroprotective effects, and their healing properties are superior to other marine organisms. Sea cucumber extract has a high concentration of vitamins A and B (B1, B2, B3), as well as significant amounts of minerals (calcium, magnesium, iron, zinc, selenium, germanium, strontium, copper, manganese). These ingredients are easily absorbed, provide hydration and at the same time stimulate the regeneration of damaged epidermal cells. According to the literature, sea cucumbers contain high amounts of collagen, which is relatively safer compared to collagen of terrestrial animal origin, as well as significant quantities of mycopolysaccharides. Approximately 70% of the insoluble collagen fibers found in the body of sea cucumbers are proteins and can be gelated when hydrolyzed.

The healing process can be enhanced by certain bioactive metabolites that contribute to tissue repair. Glucosaminoglycans in the shell tissue of *Stichopus vastus* and *Stihopus hermanni* have been studied and found to have a healing effect in rats. Among sea cucumbers, *S. hermanni* is used in traditional medicine for a variety of diseases, including wound healing [9]. In vivo animal studies show that wounds with sea cucumber extracts had better and more immediate healing. The topical application of these extracts to wounds caused in animals seems to accelerate the rate of wound contraction, which is of paramount importance for the healing phases. Furthermore, a study showed that the use of *S. hermanni*-based hydrogel as a wound patch in the management of burns led to a significant contraction of the wound on days 21 and 28 after the burn. This effect is probably due to the ability of the specific wound patch to retain the active ingredients and delay their release to the injured skin, thus acting in the next stage of the healing process. This patch is advantageous in the ability to keep the bioactive components immobilized in the hydrogel’s core for longer, ensuring controlled and prolonged release. This fact could result in enhancing the action of the embedded extract while tissue repair is carried out, and therefore, the successful interaction of the patch with the wound brings a positive result in healing at a later stage. Another species of sea cucumbers, *S. chloronotus*, appeared to be active in the early stages of healing. The antioxidant effect of the aquatic extract was shown to be 80% higher than the organic equivalent. Given that the excessive presence of free radicals has been associated with problematic healing, their neutralization by the antioxidants present in the aquatic extract contributes to the smooth healing process. In addition, the analysis of the fatty acid composition showed that the aquatic extract contains a higher amount of Docosahexaenoic acid (DHA) compared to the organic extract.

There has been a hypothesis that DHA can activate the production of inflammatory cytokines in injured areas, helping to control the infection and preparing the tissue for further repair. This is achieved by boosting phagocytosis, inducing keratinocyte migration to the extremities of the wounds, increasing fibroplastic chemotherapy and their proliferation, causing fragmentation of extracellular matrix proteins, and regulating the release of other cytokines and growth factors. In addition to DHA and EPA (eicosapentaenoic acid), the main fatty acids in sea cucumbers also interfere with the inflammatory process, inducing the production of resolvins. Resolvins inhibit Interleukin-1 beta (IL-1Β) and protectins, which block IL-1Β and Tumor necrosis factor alpha (TNF-α) production, through the pathways of Cyclooxygenase-2 (COX-2) and 5-Lipoxygenase (5-LOX). Based on studies carried out on other species of cucumbers, it was confirmed that the aquatic extract has a higher healing potential than the equivalent organic extract [8,9]. In addition, the anti-inflammatory effect of sea cucumbers was studied by incorporating their extract on Carbopol^®^ gel and its topical application in patients with diabetic foot ulcers for 12 weeks. TNF-α levels appeared to vary significantly between the beginning and 8th, 10th, and 12th weeks. It was suggested that the saponins contained in sea cucumber extract are likely to be responsible for preventing the lipopolysaccharide-induced production of TNF-α by the transfer factor NF-κB (nuclear factor kappa B), which is involved in the translation of many genes involved in inflammation [10].

#### 4.1.1. Stability of Sea Cucumbers’ Components

Thermal treatment significantly affects the moisture content of sea cucumbers. In addition, collagen content decreases when exposed to heat, as low-temperature treatment triggers enzymatic activities, leading to the conversion of collagen fibers into spirals. During the sea cucumbers’ storage, oxidation and non-enzymatic softening cause even greater collagen damage. Furthermore, heat exposure has a negative effect on the sea cucumbers’ polysaccharides and minerals [11].

#### 4.1.2. Safety/Toxicity of Sea Cucumbers’ Ingredients

A potential danger in the use of sea cucumbers is the presence of tiny plastic particles (size: 0.1 μm to 5 mm). These are widespread dangerous pollutants, with micro-fibers (MFs) being the main representative in ecosystems. As research shows, the entry of MFs into the colonial fluid of sea cucumbers takes place through the respiratory tract from the water. The mechanism of toxicity is not yet known. In a related study conducted on sea cucumbers of the species *Apostichopus japonicas*, the results showed that MFs caused changes in gene expression [12].

### 4.2. Ingredients of Sea Worms

The wound-healing potential of marine worms is examined with the prospect of being utilized as promising and alternative wound-healing agents with no side effects in the future.

#### 4.2.1. *Diopatra claparedii*

*Diopatra claparedii*, commonly known as Ruat Sarung, is a local marine worm found on the west coast of the Peninsula region of Malaysia. The unique ability shown by this species to regenerate its frontal and rear parts in cases of injury or self-acupuncture demonstrates its contribution to the healing effect. In a study conducted to demonstrate the curative capacity of *D. claparedii* aquatic extract in acute wounds in Sprague Dawley rats, ointment with concentrations of 0.1, 0.5, and 1.0% *w*/*w*, applied topically for a period of 14 days, was used. The results obtained were based on a histopathological analysis and keloid scar observation.

The best therapeutic effect appeared to be the 1% *w*/*w* ointment, as it showed a shorter healing time, while at the same time providing a relieving effect and improved collagen deposition with reduced scar formation compared to other products, such as common commercial ointments with acriflavine 0.1% *w*/*w* and oysters with seaweed extract (gamat) 15.0% *w*/*w*. In addition, the aquatic extract of *D. clarapedii* showed antibacterial action against *E. coli* and *P. aeruginosa* with a minimum inhibitory concentration (MIC) of 0.4 g/mL, while the analysis with nuclear magnetic resonance (NMR) spectroscopy revealed the presence of metabolites with healing action, such as amino acids, aromatic derivatives, organic acids, and vitamins, which justifies the use of this extract in wound healing [13].

##### Safety/Toxicity *D. clarapedii*

The absence of microbiological contamination, local irritation on the skin, as well as the quantitatively insignificant concentration of heavy metals, make this specific aquatic extract safe for use [13].

#### 4.2.2. Arenicola Marina

Hemoglobin derived from the marine red worm *Arenicola marina* (M101) appears to be effective in oxygen transport, while at the same time, it has antioxidant, anti-inflammatory, and antibacterial properties, rendering it beneficial in wound healing. Moreover, it seems that hemoglobin derived from *A. marina* has the ability to effectively transport O_2_ in vivo, without signs of oxidation, hypertension, or vasoconstriction, and does not exhibit immunological, allergic, or prothrombogenic reactions when applied, confirming its safety and effectiveness. M101 remains stable in a variety of ionic compositions and osmolarities [14].

##### Safety/Toxicity *A. marina*

The effect of the environmental pollutant nano-Zinc oxide (nZnO) on the metabolism of *A. marina* has been previously investigated. Researchers have observed a change in the metabolism of the sea worm, due to exposure to nZnO, with suppression of the metabolic activity of glyconeogenic and aromatic amino acids as well as inhibition in the action of fumarases, resulting in changes in the cycle of the tricarboxylic acid (TCA). These modifications have a negative impact on *A. marina*’s central metabolic pathways, impeding its growth and reproduction [15].

### 4.3. Ingredients of Sea Sponges

Sea sponges are invertebrate animal organisms, belonging to the genus “*Porifera*” and are characterized as the most primitive multicellular creatures that have existed for millions of years at the bottom of the marine ecosystem. They constitute a huge source of synthesis of new biomolecules while providing shelter for other organisms and inducing a multitude of marine processes. With regard to secondary metabolites from marine mushrooms, it is important to take into account the environmental conditions and the relationship between them and their associated microorganisms and phytoplankton, as some of the isolated bioactive secondary metabolites are considered to be produced by functional enzyme groups derived from the associated microorganism. These microorganisms are of utmost importance in the synthesis of new medicinal products, cosmetics, and dietary preparations, since they constitute renewable sources of products of natural origin [9,16]. The fact that marine mushrooms are repositories of a variety of marine microorganisms enlarges the horizons for the development of maritime biotechnology. This is evident because a wide variety of metabolites derived from mushrooms has similarities with bacterial and fungal natural products or is included in a category of chemical compounds derived by these microorganisms. Some reports have confirmed that certain chemical compounds, originally isolated from marine sponge extracts, have actually been biosynthesised by sponge-related microorganisms, since microbes live in the sponge mesh. Free amino acids, glucose, polyatomic alcohols, carboxylic acids, and nitrogenous heterocycles are mostly found in these organisms, according to the literature [17].

#### Safety/Toxicity of Marine Sponges Ingredients

In an evaluation carried out on the marine sponge *Chondrosia renifomis* regarding the in vitro toxicity, antioxidant activity, healing capacity, and photo-protection of the marine collagen hydrolysates (MCHs), it was found that the MSHs showed no toxicities, while they had high anti-oxidant action by inducing the neutralization of free radicals, as well as the ability to heal wounds by accelerating the proliferation phase [8,9,16]. Table 1 summarises several characteristics of sea organisms as sources of bioactive substances.

## 5. Bacteria

Marine bacteria produce several secondary metabolites to defend themselves against the hostile conditions of the oceans, which are a useful source of biologically active compounds.

There are many substances originating from marine bacteria, which are characterized by photoprotective, anti-aging, hydrating, antimicrobial, and anti-oxidation properties, e.g., alkaloids, proteins, peptides, lipids, mycosporins, mycosporine-like amino acids (MAAs), glycosides, and isoprenoids [8,9].

### Cyanobacteria

Cyanobacteria are prokaryotes photosynthetic organisms, often referred to as microalgae. They are resilient in both marine and terrestrial environments and have numerous biotechnological advantages over other organisms (plants, fungi, bacteria) due to their metabolic activity, which can trigger the production of many chemical compounds with a variety of uses in various fields (e.g., food, energy, health, bio-materials). Pigments (carotenoids and phycovillins), peptides, fatty acids, and polysaccharides derived from cyanobacteria are biologically active and can be used in the manufacture of medicines and cosmetics, as well as in wound healing, thanks to their antioxidant, antibacterial, viral, reproductive, immunomodulating, and immunostimulating properties.

Numerous extracts from cyanobacteria have been studied in recent years for their ability to heal wounds, but the mechanism of action for most chemical compounds remains unclear. The aquatic extract of *Aliinostoc* sp. PMC 882.14 showed a healing effect but did not induce the secretion of IL-6. It is claimed that the mechanism of action differs from the process observed in *Synechococcus elongatus* PMC 7942 but may be similar to the path of action of the apratyramide peptide (referred below), as it induces the secretion of growth factor [18,19].

#### Safety/Toxicity of Cyanobacteria

The possible cytotoxicity of the extracts in the HaCaT cell series (RAW 264.7) and in human PBMCs was investigated; no toxicity was found. However, in the more sensitive mouse cell series (RAW 264.7), a slight loss of viability was revealed with the use of some extracts. Overall, no cyanotoxins (microcystine, anatoxin, saxitoxin, cylindrospermopsin, nondularin) were identified in the raw extracts, and no cyanotoxin biosynthetic gene clusters (BGCs) were detected in the nine genomic complexes, suggesting that they present no risk of toxicity and are suitable for therapeutic purposes [18,19].

## 6. Parasites

The significant wound-healing potential of marine parasite extracts has been studied, yielding highly significant outcomes both in preclinical and clinical trials.

### Ceratothoa Oestroides

The isopode *Ceratothoa oestroides* is a primary hermaphrodite parasite, mainly found in the oral cavity in the *Dicentrarchus labrax* and the sea bream *Sparus aurata*, causing serious damage and often leading to the death of host organisms, especially young fish. An oily extract of *C. oestroides* recently showed significant healing ability in vivo.

Sofrona et al. found that topical application of the bioactive fraction with a sequence of separate polarity (organic and aquatic) extracts of *C. oestroides* in mouse wounds resulted in complete wound rehabilitation and restoration of the skin to almost its natural architecture, without the presence of inflammatory agents [20]. In another research [21], clinical examination, observation of the ulcer area, transdermal water loss (TEWL) results, and epidermal hydration confirmed healing properties that became apparent after 45 days of care and led to complete healing in most patients (61%). Also, researchers found that the use of innovative treatment with *C. oestroides* extract resulted in a significant reduction in the size of the ulcer over time. The readings first started at very high levels and gradually fell as the wound healed, indicating the reconstruction of the epidermal barrier. The same applies to the rate of epidermal hydration, which started low and gradually increased to almost normal levels after wound healing.

In 2019, a case study was conducted on diabetic foot ulcers in a 58-year-old obese man who was a smoker and had a history of uncontrolled type II diabetes, peripheral neuropathy, and Hodgkin lymphoma. He was treated with an ointment containing *C. oestroides* extract in olive oil for 5 months without antibiotic administration. To assess the treatment with the extract, the transdermal water loss and epidermal hydration were measured, accompanied by photographic documentation. The condition of the patient’s ulcer showed a progressive improvement in healing at each control stage of their course. Despite the patient’s concomitant conditions, which do not favor the healing process, such as immunosuppression, Hodgkin’s lymphoma, unregulated diabetes, obesity, peripheral neuropathy, smoking, and the anatomical area of the wound, the treatment had a strong healing capacity. It is worth noting that no antibiotics were required. The use of this innovative treatment with *C. oestroides* extract contributed significantly to the progressive reduction in the size of the ulcer. The transdermal water loss followed a healing pattern, starting from very high values and reaching approximately normal levels after the wound was closed, revealing the restoration of the epidermal barrier. Accordingly, the rate of skin hydration started at low levels and reached almost normal levels after healing. Diabetic foot ulcers are very difficult to manage and treat, especially in patients with other serious concomitant diseases, such as immunosuppression. Five months after starting treatment with *C. oestroides*, the patient experienced complete recovery. The blood tests that followed showed a significant decrease in inflammation markers. No microbial growth occurred in ulcer cultures, either before or after treatment. Both the clinical evaluation and the objective measures were in agreement, showing complete recovery. In conclusion, the effectiveness of the application of the ointment with extract *C. oestroides*, in this case, offers a new option for the management of diabetic foot neuropathic ulcers, especially in patients with severe concomitant diseases [6,22].

#### Safety/Toxicity of *C. oestroides*

Issues relating to potential toxicity and potential risks from the use of *C. oestroides*, as well as significant difficulties associated with successful safety monitoring, need to be further investigated and addressed [6,22].

## 7. Antioxidants of Marine Origin in Skin Wound Treatment

It is well documented that marine-derived compounds possessing antioxidative functionalities accelerate and improve wound-healing capability since they can interact in the various stages of the wound-healing process.

### 7.1. Astaxanthin

**Figure 3:**
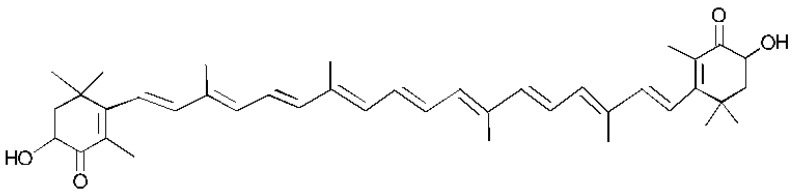
Structure of Astaxanthin.

Astaxanthin (Figure 3) is an orange-red non-vitamin A carotenoid in the Xanthophyll family. It is classified as a “pure antioxidant”, safe to use, and the natural astaxanthin has been proven to possess increased biological action when compared to the synthetic [23]. Astaxanthin is found in nature as an ingredient in bacteria, fungi, algae, carcinoids, and some species of fish. It is directly or indirectly involved in many biological pathways and has a variety of actions, including curative properties.

In a study conducted in animals in 2020, the application of a carotenoid hydrogel rich in astaxanthin revealed an increase in the content of collagen, angiogenesis, and vascularization, as well as restoration of epithelialization, resulting in the enhancement of the healing effect [24].

In 2019, the development of an innovative composition named ACF (Astaxanthin Incorporated Collagen Film) showed increased healing capacity, at 71%, in rats who underwent linear incision and full-thickness skin resection. Treatment with ACF showed improved healing in the affected area, with the presence of higher collagen content, better angiogenesis, and rapid epithelialization in a short time [25,26].

#### 7.1.1. Astaxanthin Stability

Drawbacks in the use of astaxanthin include its strong instability at high temperatures, acidic pH, oxygen, and light exposure, as well as its limited solubility in water, bioavailability, and biodisponibility. As a result, the biomedical application of astaxanthin, mainly in its pure form, is not favored. One way to address this problem is by embedding it with conventional but also innovative techniques. Its stabilization in oil, micro/nano-enzymatization, or production of nanomolecules (fat, micro-molecule, particle systems, cyclodextrins (CDs), film), has been shown to significantly improve stability, biological activity, as well as technological functionality [27].

#### 7.1.2. Safety/Toxicity of Astaxanthin

Contamination of microalgae with heavy metals must be taken into account. Due to the characteristic OH- and COO- groups on the surface of the cells of the microalgae, the absorption of positively charged heavy metals affects the growth of the microalgae and, therefore, the quality of the astaxanthin extracted from them [28].

## 8. Polysaccharides of Marine Origin in Skin Wound Treatment

Marine polysaccharides are characterized by the presence of a multitude of chemical structures, combined with important biological properties, including biocompatibility, biodegradability, anti-inflammatory, adhesive, and antimicrobial action. Table 2 shows the classification of polysaccharides by source [29].

### 8.1. Carrageenans

Carrageenans are a group of polysulphate polysaccharides of high molecular weight (MW), found in marine algae of the category *Rhodophytes*. Depending on the degree of sulfation, there are six types of carrageenans, of different solubility and source of extraction, with the most studied being the types k-, i-, and l-.

Their gelatinizing properties make them an ideal raw material for the manufacture of hydrogelated wound patches. The most studied k-carrageenans exhibit biocompatibility, hemostatic, and immunomodulatory characteristics that are essential for healing. Classified hydroglutamines, based on k-carrageenans and gelatin, show a number of advantages, as well as healing properties, compared to conventional multi-layer or networked analogs. Nanogels with therapeutic nanoparticles up to 100 μm in size, which are released depending on the temperature in the wound area (37–45 °C), as well as hydrogels created by 3D printing, which have the desired shape and specific mechanical and chemical properties are promising formulations. They are used in cases where prolonged release of antimicrobial agents, bioactive molecules, and growth agents is needed. Due to their antiviral and antibacterial properties, they have raised interest for their use as wound bioadhesives.

In fact, their bioavailability, biodegradability, and better contact with the skin are characteristics that make carrageenans preferable for use in wound patches compared to fucoidans.

Nair and colleagues [30] studied the healing properties of beta-(1-3) (1-6) glycan/carrageenan hydrogels and found that the presence of carrageenan in the composition increases the porosity of the hydrogel and activates the bonding and proliferation of fibroblasts, both in vitro and in vivo, ensuring faster wound recovery, compared to the control. In a study conducted by K. Zepon and colleagues, researchers formulated for the first time a combined “smart” wound patch, consisting of pH-sensitive hydrogel with k-carrageenan, *Ceratonia siliqua* gum, and *Cranberry* extract. The gum appeared to enhance the mechanical properties of carrageenan. The cranberry extract, rich in anthocyanins, demonstrated, in addition to antimicrobial action, a role as a marker for the pH change detection, in case of alkaline reaction, indicative of bacterial contamination in the wound fluid [31].

#### Carrageenan Stability

Researchers developed formulations with i-carrageenan and argan oil to clarify whether they affect the stability of the product, by measuring the viscosity, consistency, stability, and coherence parameters in terms of texture. It was found that the composition containing the maximum concentration of each ingredient (2% for argan oil and 1% for i-carrageenan) had the most satisfactory results, with the two ingredients acting synergistically with each other in improving the physical and chemical properties of the product, while the texture parameters of this composition were the most acceptable in the sensory evaluation [32]. In another study, formulations with k-carrageenan, sorbitol, and glycerin were developed, and the swelling and mechanical properties as well as the stability in hydrogels were examined. It turned out that the composition with the most ideal characteristics was that containing 2% k-carrageenan, 20% sorbitol, and 1.5% glycerin [33].

### 8.2. Ulvans

**Figure 4:**
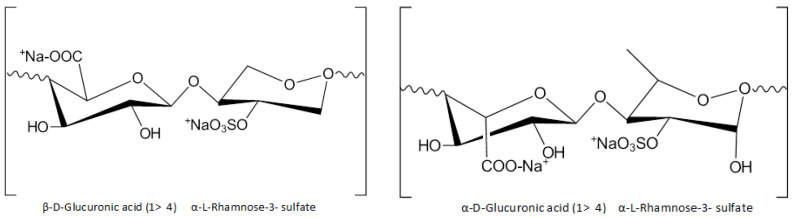
Structural variety of algae sulfur polysaccharides: main repeated disaccharides forming ulvans.

Ulvans (Figure 4) are sulfuric heteropolysaccharides that constitute one of the main biopolymers, derived from the green algae of the order Ulvales, which includes the species Ulva, *Enteromorpha*, and *Utricularia*.

Ulvans are almost insoluble in organic solvents, which creates an obstacle to the ability for chemical modifications and therefore to their widespread use in wound patches. The high pH in the solution increases the intramolecular forces in the wound area, enabling the hydrogel to acquire a high viscosity. This feature allows the modification of the gel-forming properties of polysaccharides, through changes in their structural and functional relationships. The presence of rhamnose enhances the biological action of ulvans, especially in relation to epidermal pathologies, while also improving the healing properties, reducing the attachment of bacteria to the wound, and stimulating cellular proliferation and collagen biosynthesis.

Taking into account data from studies based on experiments and models, it becomes clear that ulvans have significant antioxidant, anticoagulant, and immunomodulating biological properties both in vitro and in vivo. In addition, ulvans carry antiviral properties. Therefore, they are used as preventive agents against biofilm formation and in the manufacture of bandages for wound care and tissue restoration.

The development of a polyionic ulvan–chitosan gel complex is an example of the successful use of the physical and chemical properties of ulvans, which has shown greater stability compared to alginic acid–chitosan gel in both acidic and alkaline environments [34].

#### 8.2.1. Ulvan Stability

The presence of sulfur and carboxyl groups structurally prevents the mechanical stability of hydrogels, which is associated with active water absorption and the development of hydrolyzed degradation. In the design of wound patches, these structural characteristics of ulvans require preliminary modification so that they become insoluble and mechanical properties increase in the hydrogels. To increase their mechanical properties, an ionotropic gel of cationic polymers or non-organic additives such as boric acid, copper, calcium, zinc, or magnesium can be developed [34].

#### 8.2.2. Safety/Toxicity of Ulvans

The cytotoxicity of ulvans was assessed in cells in vitro and directly in experimental animals in vivo in a recent study. Rat lung cells, mammal cells L6, HaCaT keratinocytes, and 3T3 fibroblasts were used. The test on human cells L929, after 72 h of contact, revealed that the ulvans’ metabolism was active, without any indication of decreased viability. *Ulva* sp. ulvan extraction was found to be safe for L6 mammal cells, and even at the highest concentration (10,000 mg/mL), they showed no cytotoxicity (inhibitory concentration (IC)50 < 90 mg/mL) in 3T3 cells [35].

### 8.3. Alginate Salts

**Figure 5:**
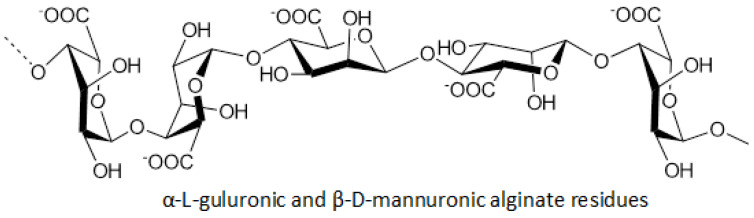
Structural variety of alginic sulfate polysaccharides: alginic segments α-L-guluronic and β-D-mannuronic acids.

Alginate salts are polysaccharides of brown algae (genus *Fucus*) and constitute one of the most common marine biopolymers. The distinctive characteristics of these metabolites form the basis for the production of nanocomposite wound patches. These acidic linear polysaccharides are distinguished by their physical and chemical properties in two types of uric acids, L-guluronic and D-mannuronic, in the form of a homogeneous or heteropolymer segment (Figure 5).

In order to create hydrogels from aqueous alginic solution, a combination is made with a cross-linked ionic agent, such as the Ca^2+^, Ba^2+^, or Co^2+^ cations, which interact with G-fractions of polymer chains. Calcium alginate wound patches high in G-fractions exchange ions (Ca^2+^-Na^+^) in smaller amounts, with exudate swelling slowly but without causing injury or pain during removal. Similar patches with a high content of M-fractions directly absorb the exudate but require special moisture during removal. Sodium ions have the ability to form transverse bonds with alginic ions, which, as porous patches, allow mechanical and targeted tissue reconstruction, acting as an almost perfect barrier film. The effective prothrombic properties, as well as the high hemostatic activity of alginates, provide the optimal moist environment in the wound area, along with good absorption of the exudate, while inducing the growth of granular tissue, reducing the concentration of pro-inflammatory cytokines, preventing the formation of free radicals, and enhancing the antimicrobial action. This fact, on a clinical level, turns into a reduction in the time required for healing and an extension of the intervals between each connection, which is carried out traumatically and painlessly. The extensive use of alginates with metallic ions in hydrophores as wound patches for the management of acute and chronic wounds, such as diabetic ulcers, traumatic wounds, and surgical incisions, as well as slope wounds, is based on other characteristics of alginates, associated with their low cost, wide availability, and high biocompatibility [36]. Examples of such wound dressings are Algicell^TM^, AlgiSite^TM^ M, and Comfeel^TM^ Plus, among others [37].

Furthermore, film and foam patches based on alginate salts seem very promising, as they appear to improve healing by normalizing gas exchange and provide protection against pollution, combined with other biopolymers, essential oils, and surfactants, which enhance dispersion. A variety of alginic-based bandages are available on the pharmaceutical market, ranging from traditional hydrogel patches to innovative lyophilic leaves and cavity wound nanogens, while combinations with Zn, Mn, Ag, glycerol, polyvinyl alcohol, and other marine polymers are also present in the formulation.

K. Murakami and colleagues [38] incorporated the healing properties of alginates together with fucoidan, chitin/chitosan, and mitomycin C into a wound patch hydrogel. The results of their experimental studies showed that this is an excellent patch, combining the chemo-electronic effect in fibroblasts and the promotion of their proliferation, along with the acceleration of re-epithelialization of tissues and granulation that occur on the seventh day. When analyzing the healing mechanisms of this patch, the combined effective action of brown algae, polysaccharides, alginates, and fucoidan is promising as it is also supplemented by the enhanced mechanical strength provided by chitin and chitosan. In cosmetic science, a combination of alginate and hyaluronic acid has been used. The hyaluronic derivative (hyaluronan) contributes significantly to healing, as it slows down the release of Ca^2+^, regulates the gelation of alginates, and provides hydration to the wound in the early stages of the healing process while activating the proliferation and migration of keratinocytes. However, despite the overall biological effectiveness of the alginate hydrogel, the difficulty of controlling the process of gel formation leads to the appearance of a heterogeneous structure and unsatisfactory mechanical strength. In an experimental model, it was noted that the healing took place faster in gel-shaped mixtures containing the alginic–hyaluronic acid combination.

At the same time, the structure in the alginate–hyaluronan hydrogel offers the possibility of releasing biologically active substances directly into the wound.

As part of the research of alginic salts as functional and bioactive materials for wound care and rehabilitation, alginate hydrogel with H_2_S was prepared as a patch and tested for morphology, degradation, substance release capacity, biological and cellular compatibility, and healing capacity. In this study, the alginate hydrogel was initially pre-treated with H_2_S, followed by characterization and evaluation of the biological properties in vitro and in vivo. The results showed that the hydrogel presented a porous structure, with pores size ranging from 50 to 100 μm, a satisfactory rate of swelling and degradation, with water absorption at 179 ± 5% of the initial dry weight within 96 h and 80% of the initial dry weight after 7 days, as well as a drug release profile beneficial for the healing process. The in vitro evaluation revealed that the optimal H_2_S concentration was 0.5% and that higher concentrations had adverse effects on cell growth and healing process, causing hemolysis and cytotoxicity. In vivo study results indicated that the highest rate of wound recovery was obtained with 0.5% alginic/H_2_S treatment. In addition, this combination appeared to induce the formation of sebaceous glands, hair follicles, and complete epithelialization, without causing inflammation or fibroplasia, as observed through histopathological observation. The exact mechanism of action remains unknown and requires further investigation [36,39].

#### Safety/Toxicity of Alginate Salts

Alginate salts, although they are biomaterials derived from brown algae and are therefore fully biodegradable, do not decompose in the body. However, they release ions and break down into smaller molecules. A chemical modification, e.g., oxidation in the chains of alginates, can contribute positively to their decomposition. Decreased mechanical properties and stiffness are possible obstacles to their use, especially in hard tissue mechanics. Changes in the chemical character can enhance the properties of the material, but also affect negatively, causing toxicity. In the framework of in vitro studies, on 3D cell culture platforms and at the level of local application, it is observed that alginate salts are a safe natural biomaterial [40].

### 8.4. Fucoidans

These are biopolymers found only in brown algae, which are classified as anionic sulfur polysaccharides. Some marine invertebrates (e.g., Japanese sea cucumbers and sea urchins) have the ability to synthesize similar polysaccharides.

Low-BB fucoidans simulate heparin and induce the production of cytokines such as HGF/SF, which plays an important role in the healing process and reepithelialization, while also stimulating angiogenesis, migration, and keratinocyte proliferation. In a study conducted by R. O’Leary and colleagues, it became apparent that certain varieties of fucoidans derived from brown algae of the genus *Fucus* actively interact with the growth factor TGF-b [41]. Fucoidans have been shown to induce proliferation, by inhibiting the action of TGF-b, thus significantly increasing the presence of fibroblasts, as well as the formation of a fibrous matrix of collagen, thereby promoting wound healing.

The healing effect of fucoidans on burn wounds was initially studied by M. Kordjazi and colleagues [42]. From the degree of activation of fibroblast proliferation (recognized as the main mechanism), collagen deposition, and the increase in the thickness of the skin, it was found that the higher the sulfur content of polysaccharides, the more intense their healing properties.

Also, as revealed in recent studies, fucoidans have a strong ability to inhibit the formation of postoperative adhesions in abdominal sores, thus being the most effective anti-adhesive and non-toxic agent, suitable for clinical use [34,41].

### 8.5. Polysaccharide from Falkenbergia Rufolanosa

*Falkenbergia rufolanosa* belongs to the *Rhodophytes*, which are found on the coasts of the Mediterranean, Australia, and New Zealand. It is an annual, self-growing red algae, extremely rich in polysaccharides, mainly sulfides.

A study was carried out examining the antioxidant ability in vitro, as well as the possible healing effects in rats with CO_2_ laser burns in vivo of FRP polysaccharide obtained from *Falkenbergia rufolanosa*, reinforced with polyvinyl alcohol (PVA) at various FRP/PVA concentrations: F1 (70:30), F2 (50:50), F3 (30:70), and PVA (100% PVA). Regarding the antioxidant action, it was revealed that the highest rate was that of the F1, which significantly promotes healing and reepithelialization after 8 days of treatment, and histological analysis found a higher content of collagen and hydroxyproline in the area of the wound, compared with the blind and PVA groups [43].

### 8.6. Pectin from Spirulina (SmP)

Spirulina is a type of cyan algae, rich in nutrients and active ingredients. The two main species of Spirulina are the cyanobacteria *Spirulina Arthrospira platensis* and *Spirulina maxima*, which have shown significant enhancement of the healing process in both in vivo and in vitro studies. The use of pectin in wound-covering products, skin protection, and scaffolds has been investigated. Recently, the successful isolation of pectin from *S. maxima* (SmP), as an active ingredient with favorable physicochemical properties, led to the search for a possible immunomodulating response of pectin in zebrafish and mouse models. In detail, a study was conducted on the effects of SmP on the in vitro proliferation and migratory activity of human dermal fibroblasts (HDFs) as well as on in vivo regeneration and curative activity in mutilated zebrafish larvae’s urea wings and on adult zebrafish skin injuries. Furthermore, the effect of SmP treatment on the transcription response of marker genes for healing was investigated. In this study, the absence of cytotoxicity was determined for the concentrations used (0.01%, 0.05%, 0.1%, and 0.5%), and the highest multiplication capacity was observed for concentrations of 0.1 and 0.05%.

Exposure to a low dose of SmP (50 μg/mL) induces regeneration in mutilated zebrafish larva wings without causing toxicity. SmP applied topically (600 μg/fish) to injured zebrafish, accelerates healing and restores the color of the skin, without causing excessive urination. The transcription response at the muscle and kidney level was in harmony with studies conducted on models from other fish and mammals. In addition, histological and transcript analysis showed that zebrafish provides an alternative model to mammals for healing and rehabilitation studies in preventive observation. These results demonstrate the potential for future use of SmP for wound healing [44].

#### Safety/Toxicity SmP

Research conducted on the cytotoxicity and cellular proliferation of raw extract from *S. platensis* used human keratinocytes. Concentrations were ranked as follows: 0.1 > 0.05 > 0.5 > 0.01% (*w*/*v*), based on cellular viability, indicating the absence of cytotoxicity, with the highest rates of cell proliferation manifested at concentrations of 0.1 and 0.05%. Furthermore, it was made clear that 50 μg/mL of raw *S. platensis* extract (methanol, ethanol, and aquatic) does not show toxicity to human fibroblasts, and in fact, the viability in cells was >80% [44].

### 8.7. Algae Polysaccharides in the Development of Wound Patches

Technology advances have contributed to algae polysaccharides’ isolation and cleansing, resulting in forming the basis for the synthesis of many types of wound patches.

Compared to traditional natural wound patches, algae polysaccharides appear to have advantages in certain physical and chemical characteristics, such as mechanical strength, emulsification, adhesive properties, hydrocolloid formation ability, and non-toxicity in terms of healing potential. In recent decades, polysaccharide hydrocarbons, i.e., three-dimensional hydrophilic polymer chains, consisting of 99% water, originating from algae, have been commonly used, both in the design of wound patches and in the reconfiguration of tissues.

These specific polymer systems are actively used in wound management, due to their high biocompatibility, low immunogenicity, and cytotoxicity, as well as their easy operation. In addition, their ability to physically and chemically modify their structure makes them an ideal material for the growth of hydrogels, which act as a natural barrier against bacteria and mimic the microarchitecture of the extra-cellular matrix of natural tissue. Their structural ability to create wound patches can be enhanced with antimicrobial and anti-inflammatory agents from ingredients such as gold, silver, copper, and zinc oxides, antibiotics, hormones, etc. The high hydrophilicity of polysaccharides makes them ideal for moisture retention in wound management. Acting directly in the healing process, they constitute an essential natural biomaterial and are used for the production of various types of wound patches [34].

## 9. Peptides/Proteins of Marine Origin in Wound Healing

Research advances concerning processes involved in wound healing as well as skin aging have led to the development of innovative products based on peptides, proteins, and growth factors to improve skin health.

### 9.1. Epinecidin-1

It is a cationic antimicrobial peptide characterized by a-elice structure and the absence of disulfide bonds and originates from the fish *Epinephelus coioides*. Seven functional uses of this peptide have been suggested: antibacterial, antifungal, antiviral, antiprotozoal, anticancer, immunomodulating, as well as healing. Recently, an evaluation of Epinecidin-1 for these functions was carried out using the HaCaT cell series, where it was shown that, at the highest concentration used, Epinecidin-1 promotes cell proliferation and cell migration in the area of the wound, while not showing cytotoxicity. Furthermore, studies in epidermal ulcers of mice and pigs from MRSA infection (Methicillin Resistant *Staphylococcus aureus*), showed that the use of the peptide contributed to the reduction of bacterial colonization around the wound, while at the same time favoring angiogenesis.

Also, the combination treatment with Epinecidin-1 and collagen brings about an improved healing effect compared to the single use of the peptide. Studies confirm that peptide increases epithelial activity, extra-cellular production of collagen compounds, and vasculation. At the same time, treatment with Epinecidin-1 in pigs has been shown to inhibit the C-reactive protein, as well as the pro-inflammatory cytokine Interleukin 6 (IL-6) [45].

#### Epinecidine-1 Stability

Heating and radiation appeared to adversely affect the antimicrobial capacity of the peptide, however, the presence of an acidic environment showed no significant effect. In fact, it was found that in acidic conditions, the antimicrobial action is maximized [45].

### 9.2. Apratyramide

**Figure 6:**
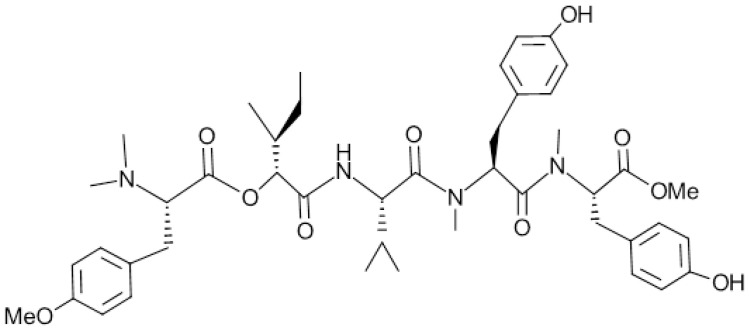
Structure of apratyramide.

Apratyramide (Figure 6) is a linear depsipeptide, i.e., a peptide in which one or more amide groups -CNHR- are replaced by the corresponding ester -COR-, isolated by the cyanobacterium *Moorea bouillonii* and enriched with portions derived from the amino acid tyrosine.

The evaluation of the in vitro examination conducted in human HaCaT cell series keratinocytes to which the specified depsipeptide was applied, showed transcription and promotion of the secretion of vascular endothelium growth factor (VEGF-A), as well as other growth factors GFs Platelet-derived growth factor-B (PDGFB) and basic fibroblast growth factor (bFGF) associated with the healing process. Thus, indirectly, there was induction of angiogenesis, which was confirmed after the application of apratyramide ex vivo, in a keratin cell layer model.

It was thus found that apratyramide provides the ability to accelerate the healing process, as well as recovery in chronic injuries [46].

### 9.3. Marine Collagen

In recent decades, marine organisms have raised interest due to collagen production. More specifically, the biomass derived from the marine organism processing industry (fish and shrimp residues, small-sized fish, and random catches such as medusas, sharks, stars, and mushrooms) creates an important but unused source of collagen. The use of discarded and underused biomass can contribute to collagen collection in a sustainable way, significantly reducing the environmental impact of this activity. The advantages and disadvantages of marine collagen are presented in Table 3 [47].

#### 9.3.1. Collagen from Fish Residues

Collagen and bioactive peptides extracted from the skin of fish, such as salmon and cod, show good water retention capacity, making them suitable for use in moisturizing formulations. Furthermore, a compound-controlled release system consisting of D microcells, L-lactide-co-glycolide acid (PLGA), fish collagen, chitosan, and chondroitin sulfate has been developed by lyophilization (freeze-drying). The amount of protein released by the system appeared to depend on the percentage of collagen in the fish. Results also indicated fibroblast proliferation and epidermal tissue reconstruction, as well as satisfactory biocompatibility [48].

#### 9.3.2. Collagen from Echinoderma

This collagen is derived from shrimp and medusas. They have a distinct connective tissue (mutable collagenous tissue—MCT), which is a source of inspiration for the synthesis of “smart dynamic biomaterials”, aimed at tissue engineering and medical rehabilitation applications. It can be used in the production of collagen barrier membranes for guided tissue reconfiguration (GRT).

#### 9.3.3. Collagen from Jellyfish

Many studies show the ability to apply in healing, sponges with collagen from jellyfish. Recently, type I collagen was extracted from *Rhopilema Esculentum*, which simulates human collagen. In a research conducted by Cheng and colleagues [49], collagen derived from jellyfish *R Esculentum* with 1-ethyl-3-(3-dimethylamine propyl)-carbodiamid (EDC) was used to create sponges with hemostatic properties, which were applied in rats with amputated tail, demonstrating, thus, the ability to use them as wound patches and haematostatic materials. Also, peptides derived from the collagen of *R. esculentum* appear to play a role in the healing process in vivo, causing an increase in the production of chemotoxic agents such as transforming growth factor beta-1 (TGFβ-1) and basic fibroblast growth factor β-FGF. Porous clusters have been prepared from collagen derived from the giant jellyfish *Nemopilema nomurai.* This collagen showed no cytotoxicity and brings upon biocompatibility with primary human fibroblasts (HF) and endothelial cells. In addition, its cellular viability was shown to be better, compared to bone-derived collagen, glucan, gelatin, and hyaluronic acid. At the same time, when implanted in vivo, it led to a similar immune response to the commercial collagen from bones. Furthermore, the production of a three-dimensional, heavily porous collagen hybrid of *N. nomurai*/haluronic acid, enabled the proliferation of fibroblasts on a large surface width, without interfering with cellular viability. In another study, crossed EDC and non-crossed collagen—both from *Rhizostoma pulmo* jellyfish—were used and they were shown to be well tolerated in vivo, with subcutaneous implantation in a rat model, similar to the collagen Mushrooms from the control group [50].

#### 9.3.4. Collagen Applications in Healing and Anti-Aging

Marine collagen exhibits significant action in the healing process. It can be used either as peptides and hydroxylates or as fibers, with structures that resemble scaffolding. The production of marine collagen peptides can be carried out by either chemical or enzymatic hydrolysis. Its molecules are more absorbable due to low MW, which increases the water solubility of collagen. In a study conducted by Wang and colleagues on rats [51], it was observed that marine collagen peptides (MCPs), derived from salmon skin, caused a noticeable improvement in traction resistance on the injured skin. There was also an increase in the rate of proliferation of fibroblasts, as well as in vascularization, 7 days after the treatment was applied to rats. In addition, the groups treated with collagen showed significantly higher levels of hydroxyproline compared to the control group, in a time- and dose-dependent manner. Similar results appeared to be observed by Zhang and colleagues [52], who also used sea collagen peptides in rats. The histological analysis showed improvement in the groups in which collagen was administered, which involved vascularization, epithelialization, and fibroblast infiltration. Both collagen deposition and growth of granular tissue and hydroxyproline, which further favors collagen deposit, showed improvement in the groups for the 7- and 11-day periods after the injury.

In conclusion, it appears that marine collagen is superior to collagen derived from mammals or bovine collagen in terms of tissue engineering, due to its biocompatibility and biodegradability.

#### 9.3.5. Marine Collagen Stability

Marine collagen is less thermal stable compared to bovine collagen, due to fewer proline and hydroxyproline residues. Its degradation temperature is <37 °C. Therefore, in order to ensure the thermal stability of marine collagen clusters, a crossover is made with chemicals such as 1-ethyl-3-(3-dimethylamine propyl)-carbodiamid (EDC), gevipine (GEN), tea polyphenol (TP), nordhydroguarietic acid (NDGA), diphenyl phosphorylazide (DPPA), and glutaraldehyde (GTA) and/or with natural processes (hydrothermal crossover). Crossing also serves to enhance mechanical strength and stiffness, delaying its decomposition [47,48,49,50,51,52,53,54].

#### 9.3.6. Safety/Toxicity of Marine Collagen

Bioavailability and cytotoxicity in collagen hydrogels were determined using NIH-3T3 fibroblasts as test cells. The viability of the cells in the tested hydrogels, with different collagen concentrations, underwent a slight decrease (*p* > 0.05) for the first two days, compared to the viability for the control group. However, after three days, a 100% increase was observed in the amount of surviving cells in the test sample. These data suggest that there is no cytotoxicity from the collagen extract, while the proliferation of fibroblasts NIH-3T3 is promoted, which can result in collagen decomposition.

As most of the efficacy studies have been carried out in vitro or on animal models, more data are needed on efficacy and possible adverse reactions on human skin.

## 10. Amino-Polysaccharides of Marine Origin in Wound Healing

Natural amino polysaccharides are exceptionally biocompatible molecules in a variety of applications such as drug delivery carriers, food stabilizers, and antibacterial agents.

### 10.1. Chitosan

**Figure 7:**
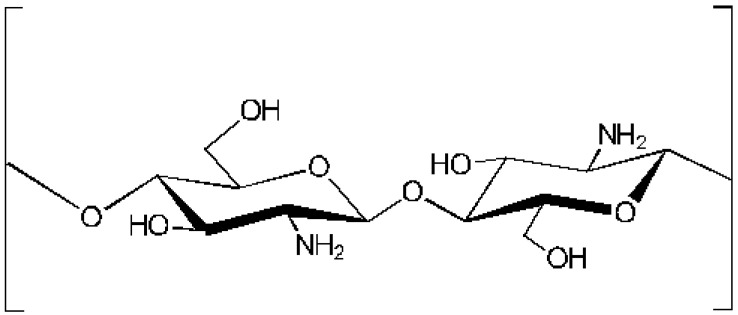
Structure of chitosan.

Chitosan (Figure 7), a derivative of chitin, is highly biocompatible and biodegradable with antibacterial and anti-cancer properties, contributing to the healing process but also to a plethora of other biological effects. Attia and El Samad [55] used chitosan hydroxide to treat wounds and found the mechanism’s almost complete recession in the healing cycle, thus revealing the development of epithelialization.

It was observed that topical application of chitosan to diabetic mice with burns accelerated the healing process while reducing the number of bacterial colonies. Therefore, it protects the burns from possible contamination, which would significantly impede healing. Sponges consisting of curcumin/chitosan/gelatin with different ratios of chitosan and gelatin were prepared and results indicated increased water absorption, antibacterial action, and healing. These sponges with higher amounts of gelatin had a faster release time of up to 4 h. Their use increased the healing effect and enhanced collagen production and wound restoration in vivo [6].

#### 10.1.1. Chitosan Stability

While chitosan is a unique and multifaceted ingredient, widely used in the fields of pharmaceuticals and biomedicine, there are limited types of products containing it (hemostatic patches, preparations for wound healing, and pharmaceutical food products—nutraceuticals). This is most likely due to the highly hygroscopic nature of chitosan, the MW variety, and the MW distribution, due to its extraction from a number of different organisms, as well as the degree of deacetylation and the level of purity. In addition, chitosan exhibits strong sensitivity to environmental factors and process conditions (heating or cooling), which leads to pressure on its structure and consequently polymer decomposition [56].

#### 10.1.2. Use of Chitosan Nanoparticles (NPs)

Within the framework of healing therapies, chitosan, as well as the aforementioned alginate salts, and their derivatives, are used as raw materials to create NP systems with excellent healing properties to provide the possibility of effective and controlled release of active ingredients.

Due to the small size of the chitosan NPs (<1000 nm), the penetration of ingredients through the epidermal tissue is favored. The strong positive charge of chitosan NPs polymers offers significant advantages, such as high synthesis stability thanks to the prevention of aggregate formation, as well as the ability to interact with negatively charged groups, e.g., cells, fungi, and bacteria. Also, solubility problems are effectively addressed with the use of chitosan NPs. The high surface/volume ratio indicates the existence of a larger surface available for interaction, which triggers the healing mechanism. This could be used therapeutically to distribute a greater amount of healing ingredients to the area around the wound.

The use of chitosan NPs appears to enhance the penetration and distribution capacity of the therapeutic compounds embedded in them. At the same time, the possible display of cellular toxicity of a substance at high concentrations is significantly limited by its incorporation into the chitosan NPs, so that the improvement in the safety of the synthesis becomes apparent [57].

#### 10.1.3. Chitosan Enriched with Silver Particles (Ag)

Hydroxyethylacrylic chitosan (HC) and sodium alginate (SA) wound patches developed with antibacterial action, thanks to the addition of silver particles (Ag). A study was conducted on the effect of the addition of Ag on HC/SA films of different densities, which were crossed with calcium. The results showed that wound patches enriched with Ag had enhanced mechanical properties and a high degree of swelling in phosphate solution. HC/SA films enriched with Ag showed antibacterial action against *E. coli* and *S. aureus*, as well as no toxicity to Vero cells. The drug release profiles from films were tested in vitro, using para-acetylamine-phenol as a soluble drug model. The increase in cross-link density and Ag enrichment led to a sustained drug release rate. It was concluded that HC/SA films enriched with Ag have a promising potential in the production of wound patches, with antibacterial properties and controlled drug release [58].

#### 10.1.4. Fucoidan–Chitosan NPs

Fucoidan acts as an obstacle to angiogenesis. The effect of MW, as well as its sulfur content, on its anticoagulant and vasogenic action is used in the inhibition of growth factors bFGF and VEGF. The importance of the weight ratio in the fucoidan–chitosan NPs in relation to the drug release profile was demonstrated. Both chitosan and fucoidan have been utilized, as mentioned above, for wound healing, with the first being applied as a patch for the proliferation and activation of inflammatory cells and the latter showing significant gel contraction, promoting the expression of integrin, as well as the activity of heparin [29].

#### 10.1.5. Safety/Toxicity of Chitosan

In general, chitosan is considered a non-toxic, biocompatible polymer, which has been approved by the authorities for a number of applications. However, it cannot be included in the generally recognized as safe (GRAS) materials, as the characteristics of chitosan, as well as the chemical modifications it undergoes, may affect its safety profile. When chitosan is included in the ingredients of a product, there is a possibility that the biodistribution profile of other components of the composition may change. Finally, the nature of these products, as well as the way they are applied, can influence the occurrence of toxicity from chitosan. Therefore, there is a need for a case-by-case safety assessment of each product, although, in the majority of cosmetic applications, its use is considered safe [59].

### 10.2. Complex Algae Salts-Polypeptides Resembling Human Elastin (HELPs)

Human elastin-simulating polypeptides (HELPs) constitute a class of bio-inspired polymers, as an alternative to animal elastin. They are synthetic, genetically coded biopolymers based on a recurring pattern of the VAPGVG hexapeptide, which is considered an excellent component for drug transport and tissue engineering due to proper cell compatibility and biocompatibility and ease in handling, design, production, and modification. In addition, HELPs, thanks to the presence of glutamine and lysine residues in the primary structure, have the ability to cross, under the influence of transglutaminase (TG), to form stable hydrogels, without the requirement of the use of harsh chemicals, such as glutaraldehyde or cross-factor analogs. A study was conducted on the combination of HELPs and alginic salts to create complexes with enhanced physicochemical properties. This research aimed at the search for the interaction between the two components, as well as the evaluation of synthesizing a personalized platform capable of transporting multifunctional agents. A series of solid polymer films based on alginic salts with different HELP concentrations were prepared, establishing a protocol and incorporating curcumin (as a model compound) into them. This is the first study to report the preparation of a bio-material based on alginate salts and HELPs for the transport of a natural product. As this research has shown, complex design is feasible, and the characteristics of the two components can be successfully integrated. The presence of HELPs in the complex played a major role, both in controlling the release of curcumin, leading to high antioxidant activity, and in preserving and possibly enhancing cell compatibility in the final product [60].

## 11. Challenges and Opportunities for Marine Origin Ingredients

The most significant challenges regarding the composition of cosmetic products with marine ingredients are related to overall sustainability, which includes production capacity, environmental toxicity, economic value, and the resulting waste.

In particular, concerning marine ingredients, it is necessary to assess the absence of heavy metals, which are normally found in the marine ecosystem, e.g., cadmium, arsenic, and mercury, as well as the lack of toxins and allergens. Moreover, marine ingredients face the issue of production volume, as they are found in small quantities and, in many cases, are difficult to isolate from the respective sources. At the same time, the issue of ensuring a stable quality of chemical elements is raised, as environmental conditions are not stable, and this leads to changes in the metabolites exported.

**Figure 8:**
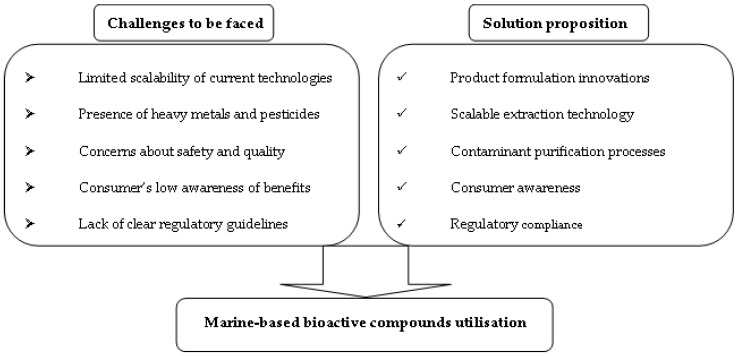
Solution proposition for addressing challenges in marine-based bioactive compounds.

To deal with the problems raised, semisynthesis is a useful strategy; the natural source of the active substance is collected and then transformed into the substance of interest. Finding sources for ingredients of marine origin meets the challenge of limited accessibility, especially when it comes to organisms living on the bottom of the oceans. However, over the years, remote-controlled submarine vehicles and modern equipment for diving have been created, thus providing the possibility of observing and sampling marine organisms living in inaccessible environments. The environmental toxicity of synthetic ingredients in personal care products and cosmetics is another concern, related to the high demand for these products worldwide. Natural ingredients have been proposed as substitutes for synthetic ingredients, as when returned to the environment, they present less risk. In addition, the intense commercialization of cosmetic products raises concerns regarding the environmental toxicity of the packaging materials used and the resulting waste, since the latter have contributed significantly to land and sea pollution. Figure 8 describes solution propositions for addressing challenges in marine-based bioactive compounds.

As far as marine environments are concerned, the main pollutant appears to be plastic, accounting for 50–90% of marine debris. Products of natural origin, produced in a sustainable way, are now used as packaging materials in cosmetics. A representative example is compostable packaging. There are also materials of marine origin that do not generate waste (zero waste), e.g., seaweed, which produce edible cosmetic packaging. These algae constitute the main example of marine packaging material, having a high content of fiber and vitamins, without the need for the use of synthetic chemicals. However, so far, algae-based packaging has only been used in the food industry [10].

## 12. Legislative Framework for Marine Components

The cosmetics field needs strict regulation, owing to its dynamic, complex, and rapid growth rate. Safety is a matter of utmost importance for any cosmetic product, regardless of the origin of its ingredients, including marine ingredients. Legislative frameworks at a global level follow a common line, but there are also significant differences.

Regarding the European market, Regulation EC No 1223/2009 constitutes the main regulatory framework to be followed by final cosmetic products. For many years, testing cosmetics on animals has been a subject of debate, as this practice has been banned in many countries. Therefore, the safety assessment of raw materials must be carried out using alternative methods. It is recommended to select in vitro test techniques, including skin and eye irritation and corrosion, mutagenicity/genotoxicity testing, and, finally, a test for photo-induced toxicity.

In the limits of the in vivo evaluation methods, a patch test of recurrent infestation in humans is used (HRIPT). The risk and exposure assessment for any chemical (macro-, micro-, or nano-grade) that enters the European market is subject to the Regulatory Authority REACH (Registration, Evaluation, and Authorization of Chemicals), in accordance with Regulation EC No 1907/2006. Regulation No. 1223/2009 sets the limits to which cosmetics manufactured must comply in order to conform with the rules of Good Manufacturing Practice (GMPs) [10].

## 13. Conclusions

Since the difficulties caused by injuries are mostly associated with treatment and management procedures that limit wound repair rather than tissue integrity restoration, several studies are aimed at achieving more efficient wound therapies, in order to reduce health costs and provide long-term relief and, ultimately, effective scar healing. To this orientation, the ¨recruitment¨ of natural active ingredients has gained great attention not only from researchers but also from the public due to the general belief that they are harmless. Especially, in recent years, the demand for biomolecules from marine resources has been exponentially on the rise due to their unique chemical and biological properties that are not found in terrestrial resources.

This review article combined recent research showing that chemical compounds from marine organisms and microorganisms have the potential to be used effectively in skin restoration during wound healing. Considering the marine resources, it has been demonstrated that the marine ecosystem, due to its high biodiversity, represents a plentiful source of bioactive compounds with diverse structures and activities. Among them, antioxidants, polysaccharides, peptides, and proteins are abundantly found in all marine organisms, serving biological and structural functions. They are often isolated from a variety of marine organisms, including algae, crustaceans, fish, shellfish, mollusks, and microorganisms, and have been extensively investigated for their potential applications in various sectors such as wound healing.

Based on the data available so far, there is a significant need for further investigation of marine ingredients in terms of safety of use and possible occurrence of toxicity, both immediately and in the long term. Further research is also needed in terms of their application and use, in order to find the most suitable cosmetic forms and/or pharmaceutical preparations to maximize the use of active substances of marine origin in wound healing. Further research for the optimal combinations of these bioactive substances is required, in order to promote the maximum healing effect in the shortest possible time, as well as the use of appropriate agents, where necessary, to ensure the maximum stability of the ingredients, controlled or prolonged release, avoidance of adverse effects/interactions, and the full potential of active chemical compounds in terms of effectiveness. Finally, it is considered necessary to establish additional protocols so that future research can be carried out with the least environmental burden and with respect for the marine ecosystem and the sustainability of the planet.

## Figures and Tables

**Table 1:** Active contents, biological effect, and safety characteristics of sea cucumbers, worms, and sponges.

Sea Organism	Active Contents	Biological Effect	Safety/Toxicity
Cucumbers	Saponins, chondroitin, sulfates, collagen, vitamins, amino acids, phenols, minerals, gelatin, triterpenic glycosides, carotenoids, trace elements, fatty acids, bioactive peptides	Anti-cancer, antioxidant, antimicrobial, anticoagulant, neuroprotective action, healing	No toxicity/safe to use in the absence of tiny plastic particles
Worms	Amino acids, aromatic derivatives, organic acids, vitamins	Healing	No toxicity/safe to use in the absence of environmental pollutants
Sponges	Amino acids, glucose, polyatomic alcohols, carboxylic acids, terpenoids, peptides, nitrogenous heterocycles	Antifungal, antimalarial, antibacterial, antiviral, antitumor, immunosuppressive, cardiovascular	No toxicity/safe to use in the absence of environmental pollutants

**Table 2:** A variety of marine polysaccharides available depending on the source.

Marine Source			
Red Algae	Brown Algae	Green Algae	Crustaceans and Marine Waste
Carragenan	Alginate	Ulvan	Chitin
Galactans	Fucoidan		Chitosan
Porphyran	Laminarin		Chitooligosaccharides
Agarose			Hyaluronans
			Chondroitin Sulfates

**Table 3:** Advantages and disadvantages of marine collagen.

Marine Collagen	
Advantages	Disadvantages
Easy availability	Low thermal stability
Easy accessibility	Low mechanical strength and stiffness
Lower costs	Rapid rate of biodegradation
(Useful) Waste is reduced	
No transmission of diseases	
Easily hydrolyzed	
No religious constraints	

## Data Availability

The authors declare that no data were used for the present work.
